# Molecular diversity of LysM carbohydrate-binding motifs in fungi

**DOI:** 10.1007/s00294-014-0471-9

**Published:** 2015-01-15

**Authors:** Gunseli Bayram Akcapinar, Lisa Kappel, Osman Ugur Sezerman, Verena Seidl-Seiboth

**Affiliations:** 1 Faculty of Natural Sciences and Engineering, Biological Sciences and Bioengineering, Sabanci University, Tuzla, 34956 Istanbul, Turkey; 2 Present Address: Research Division Biotechnology and Microbiology, Institute of Chemical Engineering, Vienna University of Technology, Gumpendorfer Strasse 1a, 1060 Vienna, Austria; 3 Research Division Biotechnology and Microbiology, Institute of Chemical Engineering, Vienna University of Technology, Gumpendorfer Strasse 1a, 1060 Vienna, Austria

**Keywords:** LysM, Fungi, Chitinase, Symbiosis, Plant defence responses

## Abstract

LysM motifs are carbohydrate-binding modules found in prokaryotes and eukaryotes. They bind to *N*-acetylglucosamine-containing carbohydrates, such as chitin, chitio-oligosaccharides and peptidoglycan. In this review, we summarize the features of the protein architecture of LysM-containing proteins in fungi and discuss their so far known biochemical properties, transcriptional profiles and biological functions. Further, based on data from evolutionary analyses and consensus pattern profiling of fungal LysM motifs, we show that they can be classified into a fungal-specific group and a fungal/bacterial group. This facilitates the classification and selection of further LysM proteins for detailed analyses and will contribute to widening our understanding of the functional spectrum of this protein family in fungi. Fungal LysM motifs are predominantly found in subgroup C chitinases and in LysM effector proteins, which are secreted proteins with LysM motifs but no catalytic domains. In enzymes, LysM motifs mediate the attachment to insoluble carbon sources. In plants, receptors containing LysM motifs are responsible for the perception of chitin-oligosaccharides and are involved in beneficial symbiotic interactions between plants and bacteria or fungi, as well as plant defence responses. In plant pathogenic fungi, LysM effector proteins have already been shown to have important functions in the dampening of host defence responses as well as protective functions of fungal hyphae against chitinases. However, the large number and diversity of proteins with LysM motifs that are being unravelled in fungal genome sequencing projects suggest that the functional repertoire of LysM effector proteins in fungi is only partially discovered so far.

## Introduction: what are LysMs?

LysMs are a family of carbohydrate-binding modules with a length of approximately 50 amino acids and bind to *N*-acetylglucosamine (GlcNAc)-containing glycans, such as chitin, chitin-like compounds and peptidoglycan (Buist et al. 2008). Binding specificities can range from general chitin-binding functions to specific recognition of substituted chito-oligosaccharides in symbiotic plant–microbe interactions. LysMs have a βααβ structure in which the two β-strands form an antiparallel β-sheet (Buist et al. 2008; de Jonge et al. 2010; Koharudin et al. 2011; Liu et al. 2012; Ohnuma et al. 2008). Proteins often contain tandem LysMs that can assemble into quaternary structures (Liu et al. 2012; Sanchez-Vallet et al. 2013).

Entries for LysMs can be found in all protein domain databases and modular protein architecture search tools, e.g. PFAM (PF01476), SMART (SM00257) and InterProScan (IPR018392). LysMs were not only first described in bacteriophages and bacteria, but also more than 95 % of proteins with LysMs currently deposited in public databases are of bacterial origin. This is also evident from the respective entry in the Carbohydrate-Active enZymes (CAZy) database (Lombard et al. 2014), where LysMs are classified as carbohydrate-binding module family (CBM) 50 (http://www.cazy.org/CBM50.html).

However, as we will show in this review, LysMs are also a prevalent protein family in fungi, which produce various proteins containing LysMs with a diversified amino acid sequence spectrum.

## Occurrence of proteins with LysMs across different kingdoms of life

The abbreviation LysM is derived from the original name lysin motif, because they were first described in bacteriophage proteins (Birkeland 1994; Garvey et al. 1986; Ponting et al. 1999) where they were found as parts of lysins, which are hydrolytic enzymes that are responsible for cleaving the bacterial host’s cell wall during the final stage of the lytic cycle. In these proteins, the LysMs anchor the enzymatic domains to the cell wall through peptidoglycan-binding (Buist et al. 2008). Subsequently, LysMs were also found in bacterial and eukaryotic proteins, and nowadays it has been established that they are commonly found in all kingdoms of life (Buist et al. 2008). In the CAZy database, proteins with LysMs (CBM 50) are listed from archea, bacteria, eukaryotes and viruses and they can be found in many different types of proteins including glycoside hydrolases, transglycosylases, peptidases, amidases, chitinases, receptor-like kinases and proteins without any other additional domains. With the increasing number of proteins with LysMs, also the variability of the carbohydrate-binding specificities of these proteins is becoming evident, and thereby defines the different biological functions of these proteins.

Several bacterial proteins containing LysMs have already been characterized in considerable detail [for details see e.g. (Buist et al. 2008)]. The first structure of an LysM domain, solved by NMR, was reported for the murein transglycosylase MltD from *Escherichia coli* (Bateman and Bycroft 2000). Among well studied bacterial proteins with LysMs are also the peptidoglycan hydrolases with *N*-acetylglucosaminidase activity AcmA from *Lactococcus lactis*, which contains three C-terminal LysMs, and AtlA from *Enterococcus faecalis*, which contains even six C-terminal LysMs (Buist et al. 2008; Eckert et al. 2006; Liu et al. 2012). It was recently shown that, unlike eukaryotic LysM domains, the LysMs of AtlA do not form any stable quaternary structure and contribute to binding in an additive instead of a coordinative manner (Mesnage et al. 2014).

In plants, LysMs are found in LysM receptor kinases, where they fulfil a fascinating range of important functions in plant–microbe interactions associated with the perception of GlcNAc-containing oligosaccharides (Gust et al. 2012; Knogge and Scheel 2006). These LysM receptor-like kinases are unique to plants and are involved in the perception of ‘friend and foe’ (Knogge and Scheel 2006). Depending on the biological context, they are able to trigger the induction of plant defence responses or symbiotic interactions. Rhizobacterial Nod factors, which are substituted chito-oligosaccharides, are important signatures for the establishment of beneficial symbioses between nitrogen-fixing bacteria and legumes (Gust et al. 2012). Symbiotic interactions between mycorrhiza fungi and plants are also mediated via LysM receptor kinases and mycorrhization factors, which are structurally similar to Nod factors and are chito-oligosaccharides modified by *N*-acylation and sulfation (Gough and Cullimore 2011; Gust et al. 2012; Op den Camp et al. 2011). In contrast to these beneficial interactions, the presence of undecorated chitin fragments or chito-oligosaccharides signals to the plant the presence of potential fungal pathogens, and in this case LysM receptor kinases are involved in plant immune activation (Gust et al. 2012; Knogge and Scheel 2006).

In fungi, LysMs are predominantly found in two types of proteins: (1) associated with catalytic protein modules. This form is mainly in chitinases, where they define the protein architecture of subgroup C chitinases, which are specifically found in the fungal kingdom (Gruber and Seidl-Seiboth 2012; Gruber et al. 2011b). (2) in LysM effector proteins, which are secreted proteins that have multiple LysMs but no catalytic domains (de Jonge and Thomma 2009). These two types of proteins with LysMs represent the overwhelming majority of LysM-containing proteins and can be found in many different fungi with all types of life styles. It should be mentioned that, depending on the fungal species, LysMs can also be found in proteins containing other functional modules, e.g. CyanoVirin-N homology (CVNH) domains, *N*-acetylmuramoyl-Lalanine amidase domains and polysaccharide deacetylase type 1 domains (de Jonge and Thomma 2009; Koharudin et al. 2011; Martinez et al. 2012) and probably this list will have to be extended with the increasing number of fungal genomes that are being sequenced.

## LysMs in fungal subgroup C chitinases

Fungi utilize chitinases not only to degrade exogenous chitin for nutritional purposes, but also to remodel and recycle the chitin in their own cell wall, where it is not the most abundant carbohydrate polymer, but an important structural component, usually located in the inner layers of the fungal cell wall, close to the plasma membrane (Ruiz-Herrera 1991). While yeast cell walls have a low chitin content of 0.5–5 % and the localization of chitin is restricted to septa, constriction rings and budding scars (Bulik et al. 2003; Chaffin et al. 1998), cell walls of filamentous fungi consist of up to 20 % or more of chitin and it can be found throughout the whole cell wall of hyphae (Ruiz-Herrera 1991). In the fungal cell wall, chitin is cross-linked to β-1,3-glucan and this glucan–chitin complex is covalently bound to other polysaccharides (Latgé 2007; Muzzarelli et al. 2012), but detailed conformational analyses of fungal chitin are technically very challenging and unfortunately not available in the literature. Extended functions of fungal chitinases include defences against other fungi and aggressive attack when parasitizing other organisms, e.g. mycoparasitism (fungi that parasitize other fungi), entomopathogens (fungi that attack insects) and nematode-trapping fungi (Seidl 2008).

Comparative genome analyses showed that fungi have highly diversified chitinolytic enzyme machineries and usually between 10 and 30 different chitinases, which belong exclusively to glycoside hydrolase (GH) family 18 (Seidl 2008). Fungal chitinases can be divided into three subgroups, A, B and C (Seidl 2008) and further extensions to this classification have been proposed to include GH family 18 proteins with different functions that have more recently been described, e.g. endo-*N*-acetylglucosaminidases (Junges et al. 2014). Subgroup C chitinases are—based on the open reading frames of their genes—large proteins of 150–250 kDa and contain multiple CBMs of families CBM 50 (LysM) and CBM 18 (chitin-binding) (Gruber et al. 2011a, b; Tzelepis et al. 2012, 2014). It has been suggested that in analogy to other CBMs in hydrolytic enzymes, these enable the attachment of the enzymes to insoluble polysaccharides, thereby enhancing the degradation of such substrates. A similar protein architecture is also found in the α-subunit of killer toxins of the yeast *Kluyveromyces lactis,* where they aid in local degradation of the host’s cell wall, subsequently allowing the ƴ subunit to enter the cell, which leads to cell cycle arrest (Mentlak et al. 2012). Biochemical characterization of fungal subgroup C chitinases has not been reported yet. However, the chitinases PrChi-A from the fern *Pteris ryukyuensis* has been studied and particularly its LysMs have already been characterized in more detail (Ohnuma et al. 2008; Onaga and Taira 2008). PrChi-A has been reported as a plant chitinase, but phylogenetic analyses revealed that this type of proteins does usually not occur in plants. Therefore, it has been speculated that the cloning of PrChi-A could have been due to a fungal contamination of the plant DNA (Zhang et al. 2009). This could, for example, also be an endophytic fungus. It should be added that another possible explanation for the source of this gene could have been a horizontal gene transfer between a fungus and the plant. However, this protein matches not only the modular architecture of fungal subgroup C chitinases, but the authors themselves also stated that its closest matches are fungal proteins (Ohnuma et al. 2008), i.e. subgroup C chitinases and, therefore, the available data for PrChi-A are of particular interest for further characterizations of this group of chitinases and fungal LysMs.

## LysM effector proteins

Another type of proteins that occur only in fungi is LysM effector proteins (de Jonge and Thomma 2009). LysM effector proteins are usually secreted proteins and contain multiple LysMs but no catalytic domains. Several LysM effector proteins were already shown to be involved in the dampening of host responses of plants against pathogenic fungi and their protection against chitinases. The first fungal LysM effector protein that was described was Ecp6 from the tomato pathogen *Cladosporium fulvum* (*Passalora fulva*) (Bolton et al. 2008; de Jonge et al. 2010). Ecp6 is a secreted protein that contains three LysMs. It binds chito-oligosaccharides that are released from the fungal cell wall by plant chitinases and thereby prevents the detection of these chito-oligosaccharides by plant proteins, i.e. LysM receptor-like kinases. Therefore, Ecp6 masks the presence of the fungus and was shown to be an important factor for fungal pathogenicity. Ecp6 has a high affinity for chito-oligosaccharides of various lengths, whereas Avr4, a chitin-binding lectin from *C. fulvum*, which does not contain LysMs, binds preferably to polymeric chitin. Thereby, it protects fungal hyphae against chitinases, whereas Ecp6 does not exhibit this property (de Jonge et al. 2010; van den Burg et al. 2006).

In the rice blast fungus *Magnaporthe grisea,* the LysM effector protein Slp1, which contains two LysMs, binds also to chito-oligosaccharides and is able to suppress chitin-induced plant immune responses, including generation of reactive oxygen species and plant defence gene expression (Mentlak et al. 2012). Slp1 accumulates at the interface between the fungal cell wall and the rice plasma membrane. Slp1 has three *N*-glycosylation sites and that simultaneous *N*-glycosylation of each site by the α-1,3-mannosyltransferase Alg3 was found to be required to maintain protein stability and the chitin-binding activity of Slp1, which are essential for its effector function (Chen et al. 2014).

In the hemi-biotrophic wheat pathogen *Mycosphaerella graminicola,* three genes encoding LysM effector proteins are present in the genome, referred to as Mg3LysM, Mg1LysM, and MgxLysM (Marshall et al. 2011). *Mg3LysM* and *Mg1LysM* genes were strongly expressed during symptomless leaf infection. Mg3LysM contains—similar to Ecp6—three LysMs, while Mg1LysM and MgxLysM have only one. Both proteins, Mg3LysM and Mg1LysM, bind chitin, but only Mg3LysM blocked the elicitation of chitin-induced plant defences. Interestingly, both Mg1LysM and Mg3LysM also protected fungal hyphae against plant-derived hydrolytic enzymes in contrast to *C. fulvum* Ecp6. While *Mg1LysM* deletion mutant strains of *M. graminicola* were fully pathogenic toward wheat leaves, *Mg3LysM* mutant strains were severely impaired in leaf colonization and did not trigger lesion formation. Further, Mg3LysM deletion strains were unable to undergo asexual sporulation, which in *M. graminicola* takes place during the necotrophic growth phase on leaf tissue (Marshall et al. 2011). Mg1LysM and Mg3LysM could also be distinguished by their ability to diminish chitin-induced defence response activation of tomato cell cultures when added simultaneously with the elicitor (Lee et al. 2014). The presence of 100 nM Mg3LysM was able to strongly inhibit the rapid (less than 10 min) alkalinization of the culture medium following the addition of 10 nM (GlcNAc)_6_. These data are in analogy to what has been observed for Ecp6 (de Jonge et al. 2010). In contrast, 100 nM Mg1LysM had no effect on the ability of chitin to induce this defence response, suggesting that Mg1LysM is less able to prevent the recognition of chitin by tomato cells (Lee et al. 2014).

LysM (effector) proteins can also be found in fungi that have other life styles including many saprotrophic fungi, mycoparasites and dermatophytes (de Jonge and Thomma 2009; Gruber et al. 2011b; Martinez et al. 2012). The protein Tal6 from the mycoparasite *Trichoderma atroviride* contains as many as seven LysMs and the respective gene is located next to a subgroup C chitinase gene (*tac6*) (Seidl-Seiboth et al. 2013). This genomic organization was also detected for other *tal*-genes encoding LysM proteins in *Trichoderma* and related species (Gruber et al. 2011a, b). Tal6 inhibited spore germination of *Trichoderma* spp. but not of other fungi (Gruber and Seidl-Seiboth 2012). Therefore, this protein rather showed an inhibitory than a protective effect, which again expands the repertoire of functions of fungal LysM effector proteins. Tal6 contains seven LysMs, of which four are highly similar to each other, whereas the other three show considerable sequence variability. The germination-inhibiting function of Tal6 was dependent on the presence of all seven LysMs as a truncated protein version containing only the four conserved motifs exhibited chitin-binding but not germination-inhibiting properties (Seidl-Seiboth et al. 2013). It should be noted that this biological function can be regarded as the opposite of a protective function against chitinases that was reported for other fungal LysM effector proteins. Tal6 was also tested for protective activities in analogy to the experiments performed with other LysM effector proteins but these were not detected.

Comparative genome analysis of the dermatophyte *Trichophyton rubrum*, the major cause of athlete’s foot disease, and related dermatophytes, revealed that the genomes of these fungi are strongly enriched for gene families containing LysM proteins (Martinez et al. 2012). The number of genes containing LysMs ranges from nine in *Trichophyton verrucosum* to 31 in *Microsporum canis*. The predominant types of protein architecture for proteins with LysMs in these fungi were those of subgroup C chitinases and LysM effector proteins (Martinez et al. 2012). None of these LysMs was functionally characterized yet, but it will be interesting to study their functions and properties in more detail.

## Carbohydrate-binding specificities of fungal LysMs

LysMs are generally regarded as chitin- and peptidoglycan-binding protein modules. Although such a broad definition of the binding spectrum of carbohydrates is certainly valid when the whole protein family is considered, it should be taken into account that the binding spectrum of individual LysM proteins or motifs is in many cases very specific. This specificity is essential for the biological processes of these proteins, e.g. Nod-factor reception, and probably also for several other, so far unknown biological roles of LysM-containing proteins.

Both the Mg3LysM and Mg1LysM proteins, as well as Ecp6, produced in *Pichia pastoris*, bound chitin beads and crab shell chitin, but neither bound chitosan, xylan or cellulose although the biological effects of Mg1LysM and Mg3LysM with respect to the induction of plant defence responses are very different (Marshall et al. 2011). Only Mg3LysM was able to prevent plant-induced medium alkalinization and the gene knockout strain was impaired in the development of disease symptoms in plants. Slp1 also specifically co-precipitated with insoluble crab shell chitin and chitin beads, but not with any other tested cell wall polysaccharides, including chitosan (deacetylated chitin) and the plant cell wall polysaccharides cellulose and xylan (Mentlak et al. 2012). For Tal6, which was also produced in *P. pastoris*, only binding to chitin beads (now called chitin resin by the supplier New England Biolabs) was detected. Tal6 did not bind to any other forms of commercially available polymeric chitin. Interestingly, binding to chitosan was also observed for Tal6 (Seidl-Seiboth et al. 2013). For a truncated form of Tal6, containing only the four highly similar LysMs, binding to colloidal chitin could be demonstrated, but not for the full-length protein, which has in addition three LysMs with strong sequence variability. Furthermore, the truncated version of Tal6, but not the full-length protein, also bound to *E. coli* cell walls. These findings demonstrate the variability of binding specificities among fungal LysMs that are most likely responsible for their different biological functions, as the full-length protein, in contrast to the truncated protein, also inhibited spore germination of *Trichoderma* species. Using isothermal titration calorimetry (ITC), for Tal6 binding to chito-oligosaccharides was not detected, but ITC and surface plasmon resonance (SPR) experiments showed that both Ecp6 and Slp1 have a high affinity for chito-oligosaccharides (de Jonge et al. 2010; Frischmann et al. 2013; Mentlak et al. 2012). This is in agreement with their biological functions, where their role is to bind these short-chain carbohydrates, which are presumably released from the fungal cell wall by e.g. hydrolytic enzymes, to prevent their detection by the respective plant receptors. This is a typical example of the arms race between plants and pathogenic fungi.

It was already suggested previously that Slp1 has the capacity to form homo-dimers to provide an additional means of shielding bound chitin oligosaccharides or as a means of increasing its space-filling potential in the narrow apoplastic space around invasive hyphae, thereby enhancing its competitive inhibition of the chitin host receptor CEBiP (Mentlak et al. 2012).

In analogy to the suggested dimerization of Slp1, Ecp6 was found to dimerize during purification steps as well as in protein crystals (Sanchez-Vallet et al. 2013). Structural analysis of the LysM effector Ecp6 revealed interesting details about the binding mechanism of this protein, which has different binding affinities for chito-oligosaccharides within the individual LysMs. Ecp6 has three LysMs, and LysM1 and LysM3 cooperate to bring two chitin-binding regions together, thereby forming a deep chitin-binding groove in which one chitin tetramer is nearly completely buried. This groove binds chito-oligosaccharides with ultra-high affinity in the pM-range. The remaining LysM2 motif of Ecp6 binds chitin with lower affinity in the µM range, but can nevertheless still perturb chitin-triggered immunity of plants. It was, therefore, suggested by the authors that LysM2 may be involved in perturbation of the activation of chitin-triggered immunity by preventing the plant immune receptor dimerization that is required for the activation of immune signalling (Sanchez-Vallet et al. 2013).

The rice blast fungus *M. oryzae’s* genome encodes a protein, MoCVNH-LysM, containing a type III CVNH lectin, in which an LysM domain is inserted between individual repeats of a single CVNH domain (Koharudin et al. 2011). Such proteins were also found to be encoded in other ascomycete fungi, predominantly in plant disease-causing species. The NMR solution structure and NMR titrations for the determination of binding specificities of this protein showed that each domain behaves as an isolated unit without any inter-domain communication. The optimal binding was obtained with a (GlcNAc)_5_. The binding affinities of MoCVNH-LysM were about three orders of magnitude higher than those observed for PrChi-A LysM for (GlcNAc)_5_. This was attributed possibly to a loop region between helix α2 and strand β2, the site of largest variation in all LysM sequences (Koharudin et al. 2011), for which larger conformational changes upon substrate binding, consistent with much tighter binding, were found. In PrChi-A, the LysM domains are very resistant to thermal denaturation and this resistance was dependent on the presence of disulphide bonds. The stoichiometry of (GlcNAc)_n_/LysM domain binding was 1:1 (Ohnuma et al. 2008). (GlcNAc)_5_ titration experiments, monitored by NMR spectroscopy, allowed the identification of residues that are critical for (GlcNAc)_5_ binding. The binding site is a shallow groove formed by the N-terminal part of α1, the loop between β2 and α1, the C-terminal part of α2, and the loop between α1 and β2.

## Transcriptional profiles of genes encoding LysM proteins in fungi

In plant pathogenic fungi, the genes encoding the so far characterized LysM effector proteins are specifically upregulated during the plant–pathogen interaction. Ecp6 was first described in a proteomic approach targeting fungal proteins secreted during *C. fulvum*–tomato interactions and its gene was found to be strongly upregulated during infection (Bolton et al. 2008). *Mg3LysM* and *Mg1LysM* genes were strongly transcriptionally up-regulated specifically during symptomless stages of the leaf infection by *M. graminicola* (Marshall et al. 2011) and *spl1* from *M. grisea* was also identified based on a search for genes encoding putatively secreted gene products that were upregulated during biotrophic growth compared with growth in axenic culture (Mentlak et al. 2012; Mosquera et al. 2009).

In contrast to that, *T. atroviride* *tal6* has a completely different expression profile. It is expressed during hyphal network formation, growth on chitin and cell walls of *Botrytis cinerea* and was found to be co-regulated with the GH family 18 gene *tac6*, which is genomically located next to *tal6*. *T. atroviride* and *T. virens* are both mycoparasites, but differ in their abilities to directly interact with plants, where *T. virens* is much more efficient than *T. atroviride* (Gaderer et al. 2014; Vargas et al. 2008). To analyse whether genes encoding LysM proteins are expressed during the interaction of *T. virens* with plants, their gene expression was tested in biomass harvested from mycelial–root interactions of *T. virens* with maize challenged with the pathogen *Cochliobolus heterostrophus*. The results showed that in this pathosystem, the expression of *tvl1*–*tvl6* was even lower than in mycelia with glucose as carbon source (Kappel L. and Seidl-Seiboth V., unpublished data).

## Diversity of proteins with LysMs in fungi

Although the range of different functions of fungal LysMs is only partially understood yet, it is evident that proteins containing LysMs are a ubiquitous feature of fungi although sequence comparison of fungal LysMs showed so far very few overall conserved residues (de Jonge and Thomma 2009; Gruber et al. 2011b; Martinez et al. 2012). The consensus patterns in PFAM and SMART are currently mainly based on bacterial LysMs, as can be seen in the respective database entries. This is probably not only because LysMs were first described in these organisms, but also because more than 95 % of proteins with LysMs currently deposited in public databases are of bacterial origin. To get a better understanding of the molecular diversity of LysMs in fungi, the phylogenetic relationships of fungal LysMs and underlying consensus patterns and their structural features need to be considered in more detail.

*Trichoderma* and dermatophytic fungi differ from the standard PFAM hidden Markov model (HMM) and contain three prominent cysteine residues not present in the standard PFAM profile (Gruber et al. 2011b; Martinez et al. 2012). Previous reports on eukaryotic LysM modules suggested that disulphide bond formation is a posttranslational modification essential for carbohydrate recognition, but usually the respective cysteines are found between the LysMs, e.g. the CXC motif present in the Nod-factor reception protein (NFP) from *Medicago truncatula* (Lefebvre et al. 2012) and the chitin elicitor receptor kinase 1 (AtCERK1) from *Arabidopsis thaliana* (Liu et al. 2012). A notable exception is the chitinase PrChi-A from the fern *P. ryukyuensis*, which, as mentioned above, has a protein architecture that fits perfectly to fungal subgroup C chitinases. However, the LysMs from PrChi-A contain—as has been described for other fungal LysMs—cysteine residues which were already reported to form disulphide bridges within the LysMs of PrChi-A (Ohnuma et al. 2008). Interestingly, not all fungal LysMs appear to have this protein signature, as e.g. Ecp6 and Slp1 have again cysteines between the LysMs, but not within them, which were already confirmed to form disulphide bridges in Ecp6 (Sanchez-Vallet et al. 2013).

**Fig. 1:**
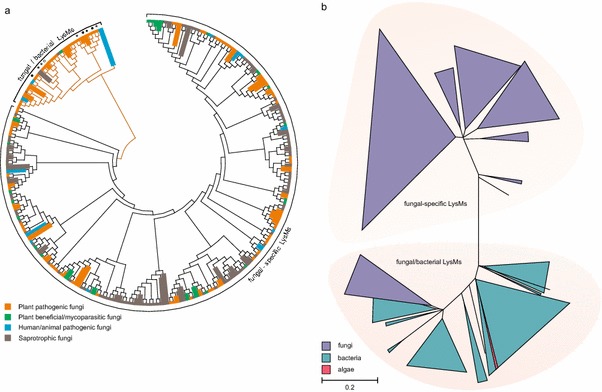
Phylogenetic analysis of fungal LysM motifs. **a** Phylogenetic analysis of fungal LysMs extracted from the MycoCosm JGI genome portal (Grigoriev et al. 2014). The following fungal genomes were analysed: *Neurospora crassa, Aspergillus nidulans, A. fumigatus, A. niger, Trichoderma reesei, T. atroviride, T. virens, Nectria haematococca* (*Fusarium solani*)*, Mycosphaerella graminicola, Phycomyces blakesleeanus, Cochliobolus heterostrophus, Batrachochytrium dendrobatidis, Phanerochaete chrysosporium, Laccaria bicolor, Postia placenta, Botrytis cinerea, Candida albicans, Fusarium graminearum, F. oxysporum, Magnaporthe grisea, Sclerotinia sclerotiorum* and this set was amended with *Cladosporium fulvum* Ecp6. LysM motifs were aligned using ClustalX (Thompson et al. 1997). Evolutionary analyses were conducted in MEGA6 (Tamura et al. 2013) using the Neighbor-Joining method. The evolutionary distances were computed using the Poisson correction method and are in the units of the number of amino acid substitutions per site. Interior-branch test with 1,000 bootstrap replications was performed and only branches with a confidence probability greater than 95 % are shown. Within the fungal/bacterial clade of LysMs, *stars* indicate the position of the three LysM domains of *C. fulvum* Ecp6, *diamonds* correspond to the two Slp1 LysMs of *M. grisea* and *black* and *white circles* indicate the LysMs of Mg3LysM and Mg1LysM of *M. graminicola*, respectively. **b** Phylogenetic analysis of LysMs derived from a protein BLAST search using the 5th LysM of Tal6, the 1st LysM of Ecp6 and the 1st LysM of Slp1 as queries. Alignment and evolutionary analysis of LysMs was performed as described for (**a**)

Thus, these data indicate that in fungi two classes of LysMs might exist, those with and without cysteine residues within the LysMs. To characterize these two classes, 250 LysMs based on the respective PFAM pattern PF01476 from fungal genome databases of various ascomycota and basidiomycota were extracted, aligned and an evolutionary analysis was performed for this review. The results (Fig. 1a) confirmed the individual observations reported before in the literature and showed that more than 80 % of those LysMs contained several cysteines at conserved positions. The other 20 % contained either no cysteine or only one—at a different position than in the other 80 % and formed a separate clade that branched off from the other sequences at a basal position of the phylogenetic tree (Fig. 1a). Therefore, fungal LysMs can be phylogenetically separated into different clades based on their aa-sequences and these differences are reflected by a characteristic cysteine pattern signature.

Interestingly, LysMs from fungi with different taxonomic relationships, as well as different life-styles, are not restricted to individual clades of the phylogenetic tree (Fig. 1a) and many fungi—including *Trichoderma* spp.—have both types of LysMs, those with and without cysteines.

LysMs with multiple cysteines appear to be more prevalent in fungi than those without cysteines. A protein BLAST search in public databases using Tal6 and Ecp6 as representatives for the two types of fungal LysMs supports this notion. Using Tal6 or individual LysMs of Tal6 as query yields exclusively fungal proteins, whereas a search with Ecp6, Slp1 or individual motifs of these proteins yields fungal and bacterial proteins. Phylogenetic analysis of the LysMs from this set of proteins shows again a large group of fungal-specific clades containing those LysMs with multiple cysteines (similar to Tal6). This branch exhibits a rather large evolutionary distance to a group of several clades with either bacterial or mixed bacterial/fungal LysMs, which contain the LysMs with none or only one cysteine, e.g. those of Ecp6, Slp1 and MgLysM3 (Fig. 1b; see figure legend for details on method and statistics).

Together, these data, in agreement with previous reports in the literature, show that fungal LysMs in fungi can be divided into two classes: a fungal/bacterial subclass and a fungal-specific class. It remains to be elucidated whether fungal-specific LysMs have distinct carbohydrate-binding specificities, e.g. preferred binding to various chain lengths, various degrees of deacetylation, or substituted chito-oligosaacharides. Further, functional studies will be needed to address whether the large reservoir of fungal-specific LysMs found in the genomes of fungi with various life styles has distinct biological functions or whether these features overlap between fungal-specific and fungal/bacterial LysMs.

## Features of the fungal-specific LysMs

**Fig. 2:**
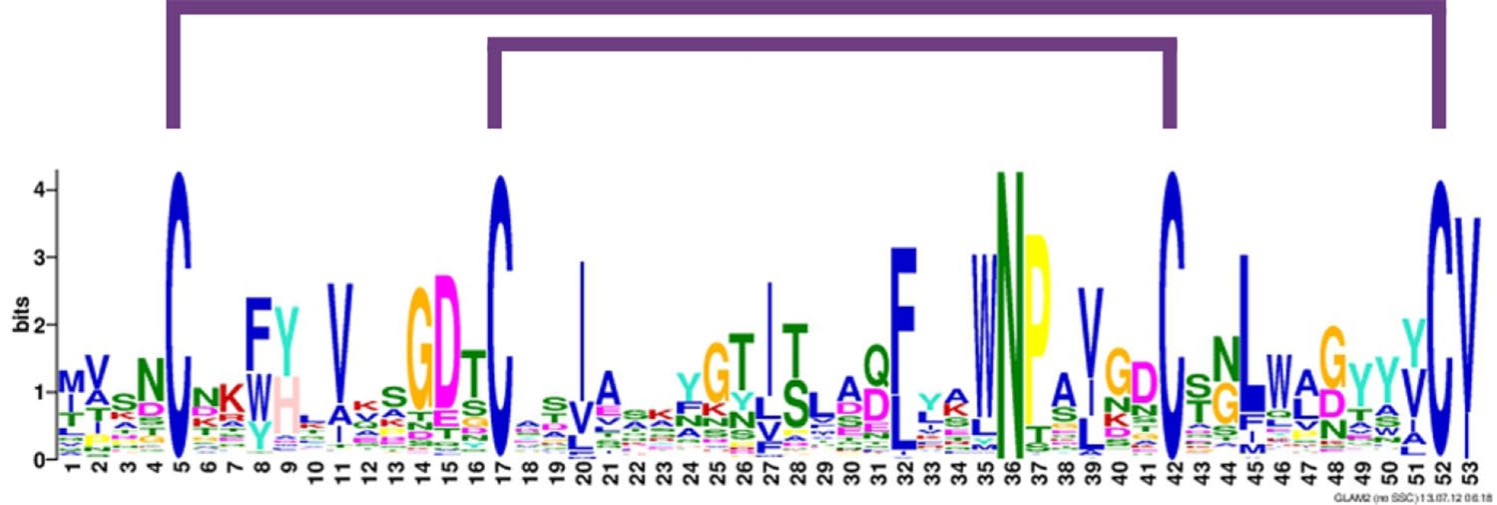
Consensus pattern of fungal-specific LysM motifs. Gapped local alignments of fungal LysM motifs and consensus patterns were generated with GLAM2 and GLAM2SCAN (Frith et al. 2008; http://meme.sdsc.edu). Using a consensus pattern generated from *Trichoderma* LysM motifs as query, fungal proteins matching the obtained consensus pattern were retrieved from the ProFASTA database [http://www.bioinformatics.nl/tools/profasta/; (de Groot and Brandt 2012) using c[^c]{8,13}c[^c]{15,17}n[^c]{6}c[^c]{8,10} as search string]

Previous reports on LysMs from *Trichoderma* (Gruber et al. 2011b) and dermatophytes (Martinez et al. 2012) revealed in both cases three conserved cysteine residues in the stretch of the LysMs matched to the length of the PFAM pattern. Visual inspection of the protein sequence of *T. atroviride* Tal6 shows that the similarity of the LysMs extended by an additional 14 aa at the N-terminus of these motifs beyond the sequence that is covered by the PFAM and SMART database consensus patterns. This extension would contain a fourth C-residue that would enable the potential formation of two disulphide bridges in these LysMs from *Trichoderma* spp. Using this elongated motif as a starting point, a general consensus pattern was derived from more than 900 fungal-specific LysMs (Fig. 2). The results showed a 53-aa consensus pattern for fungal-type LysMs with several conserved positions that are not represented in the more general LysM patterns in the SMART and PFAM databases. The most conserved positions of the fungal LysM consensus pattern are the four conserved cysteines (positions 5, 17, 42 and 52) and an N in a WNP motif at positions 35–37. The Asn located within this WNP motif is strongly conserved, even in bacterial and plant proteins (Buist et al. 2008). It is located at position 32 in motif 1LysM of AtlA, an autolysin involved in cell division in the opportunistic bacterial pathogen *E. faecalis*, but its mutation to Ala was—in contrast to other mutations in this particular LysM—not found to be critical for GlcNAc_5_ binding (Mesnage et al. 2014).

**Fig. 3:**
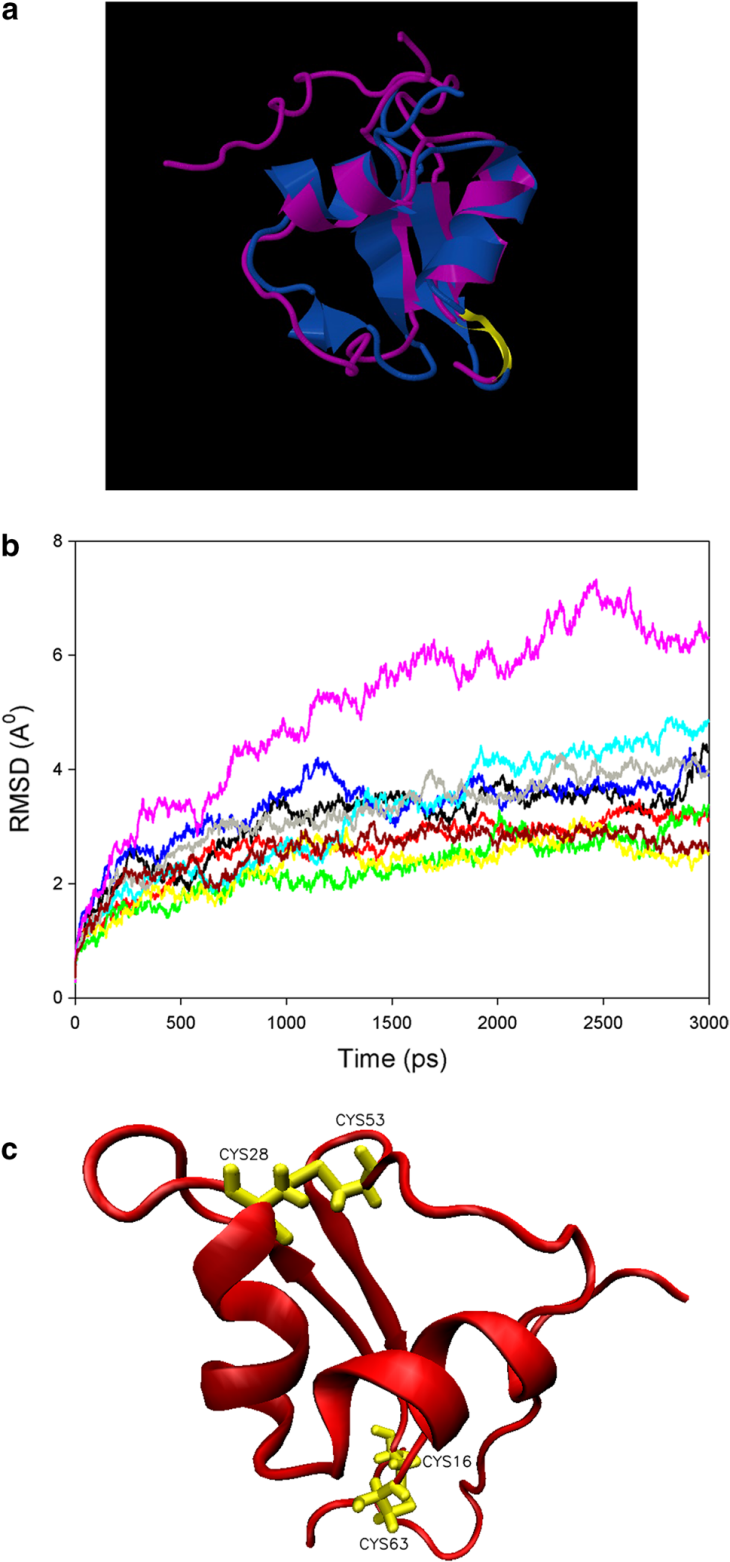
Structural modelling and molecular dynamics simulations of Tal6-5LysM. **a** Superimposed structures of the homology modelling template MltD (PDB ID:1E0G) (*purple*) and the modelled structure Tal6-5LysM (*pink*) with Z-score 3.89, RMSD 2.49 A and sequence similarity of the superimposed region 42 %. Models were constructed using the software Modeller 8v2 (Sali and Blundell 1993). The superimposed structures were created using the jCE algorithm for Combinatorial Extension (http://source.rcsb.org/jfatcatserver/index.jsp). 50 % of the substrate binding loop, between strand1 and helix1, are conserved and this region contains the ligand-binding residue Asp. The predicted substrate binding loop is highlighted in *yellow.* **b** RMSD of Cα residues along 3 ns simulations at 300 K. *Black line* Unpatched model, *Red line* CYS16–CYS28, *Green line* CYS16–CYS53, *Yellow* *line* CYS16–CYS63, *Blue line* CYS28–CYS53, *Pink line* CYS28–CYS63, *Cyan line* CYS53–CYS63, *Grey line* CYS16–CYS28:CYS53–CYS63, *Dark red line* CYS16–CYS63:CYS28–CYS53. **c** TAL6-5LysM structure at the end of 3 ns MD simulation at 300 K containing two disulphide bridges between Cys16–Cys63 and Cys28–Cys53. Cysteines are indicated in *yellow*. The numbering of the cysteines corresponds to their position in the amino acid sequence of Tal6-5LysM. For MD simulations, the NAMD/VMD software package was used (Phillips et al. 2005). The initial structure was modified by patching the possible disulphide bridge combinations using psfgen script of the VMD program. The Solvate program of the VMD package and TIP3P water were used for the water box. A 2 fs timestep was used and data collection was performed at every 2 ps. Structures were minimized 50,000 steps initially using conjugate gradient method and then equilibrated for 1 ns at 300 K. MD simulations were performed for each single patch, double patch and unpatched model in 6 A° water box using periodic boundary conditions. Additionally, 9 ns MD simulations for minimized and equilibrated structures of each model were performed at 300 K using the same method. Coordinates of Cys16–Cys28, Cys16–Cys63, Cys53–Cys63 and Cys16–Cys63:Cys28–Cys53 at the end of 9 ns simulations at 300 K were used as initial structures for equilibration steps of MD simulations at 550 K for 1 ns. Then, the equilibrated structures were simulated for an additional 1 ns at 550 K. RMSDs and radius of gyration of each simulation were calculated from the trajectory files using VMD. Total energy of the system along the simulation was extracted for each model from log files generated during the simulations using VMD and moving averages of the simulations were calculated for each model. For 3 ns MD simulations, the averages of three simulations were calculated. RMSD and total energy values for each simulation were compared

The four conserved cysteines that were detected in fungal-specific LysMs suggest that these motifs might be stabilized by disulphide bridges. This is in agreement with the LysMs from PrChi-A, where experimental evidence for disulphide bridge formation was already reported (Ohnuma et al. 2008). Structural modelling of the 5th LysM of Tal6 (Tal6-5LysM), using the LysM of the transglycosylase D (MltD) from *E. coli*) as template, also showed the typical βααβ structure of LysMs (Fig. 3a). Interestingly, the similarity of Tal6-5LysM with the LysM domain of MltD was with 23 % identity, higher than that with the LysM domain of the fungal protein MoCVNH-LysM from the rice pathogen *M. grisea*, which exhibited only 12 % amino acid identities. Tal6-5LysM contains four cysteines and molecular dynamics (MD) simulations of the homology modelled structure at 300 K on disulphide bridge formation indicated that the presence of one and two disulphide bridges stabilizes the protein (Fig. 3b). MD simulations for 9 ns and additional MD simulations at 550 K for 1 ns were also performed to further verify this stabilization. RMSD and radius of gyration values, which are a measure of the size and compactness of the simulated system, indicated that the model containing two disulphide bridges was the most stable along the course of the simulation. TAL6-5LysM structure at the end of 3 ns MD simulation at 300 K containing two disulphide bridges between Cys16–Cys63 and Cys28–Cys53 is shown in Fig. 3c. Therefore, based on the available structural data, it can be assumed that the two disulphide bridges that are formed between the four cysteines in fungal-type LysMs span Cys1:Cys4 and Cys2:Cys3 (Fig. 2).

## Conclusions

Although the data from plant pathogenic fungi enabled exciting insights into the functions of LysM effector proteins in plant–pathogen interactions and their structural and biochemical properties, it needs to be acknowledged that fungi have a wide spectrum of diverse LysM effector proteins. Considering the different life styles of those fungi, it is likely that LysM proteins have additional biological functions and fine-tuned carbohydrate-binding properties still remain to be uncovered for this protein family. The classification of fungal LysMs into a fungal-specific subclass and a fungal/bacterial subclass based on their amino acid profiles and cysteine patterns, introduced in this review, provides a basis for a more systematic view of this protein family to aid in choosing representative candidates for further analyses. This will hopefully lead to additional insights into the potential functional differentiation and carbohydrate-binding specificities of these proteins and complement our understanding of the functional spectrum of LysMs in fungi.
